# Relationships of computed tomography-based small vessel indices of the lungs with ventilation heterogeneity and high transfer coefficients in non-smokers with asthma

**DOI:** 10.3389/fphys.2023.1137603

**Published:** 2023-03-01

**Authors:** Kaoruko Shimizu, Hirokazu Kimura, Naoya Tanabe, Shotaro Chubachi, Susumu Sato, Masaru Suzuki, Kazuya Tanimura, Hiroaki Iijima, Akira Oguma, Yoichi M. Ito, Nobuyasu Wakazono, Michiko Takimoto-Sato, Machiko Matsumoto-Sasaki, Yuki Abe, Nozomu Takei, Hironi Makita, Masaharu Nishimura, Satoshi Konno, Hi-CARAT investigators

**Affiliations:** ^1^ Department of Respiratory Medicine, Faculty of Medicine, Hokkaido University, Sapporo, Japan; ^2^ Department of Respiratory Medicine, Graduate School of Medicine, Kyoto University, Kyoto, Japan; ^3^ Department of Medicine, Division of Pulmonary Medicine, Keio University School of Medicine, Tokyo, Japan; ^4^ Department of Respiratory Medicine, Nara Medical University, Kashihara, Japan; ^5^ Department of Respiratory Medicine, Tsukuba Medical Center Hospital, Tsukuba, Japan; ^6^ Data Science Center, Promotion Unit, Institute of Health Science Innovation for Medical Care, Hokkaido University Hospital, Sapporo, Japan; ^7^ Hokkaido Medical Research Institute for Respiratory Diseases, Sapporo, Japan

**Keywords:** asthma, computed tomography, pulmonary small vessels, ventilation heterogeneity, Kco

## Abstract

**Background:** The mechanism of high transfer coefficients of the lungs for carbon monoxide (Kco) in non-smokers with asthma is explained by the redistribution of blood flow to the area with preserved ventilation, to match the ventilation perfusion.

**Objectives:** To examine whether ventilation heterogeneity, assessed by pulmonary function tests, is associated with computed tomography (CT)-based vascular indices and Kco in patients with asthma.

**Methods:** Participants were enrolled from the Hokkaido-based Investigative Cohort Analysis for Refractory Asthma (Hi-CARAT) study that included a prospective asthmatic cohort. Pulmonary function tests including Kco, using single breath methods; total lung capacity (TLC), using multiple breath methods; and CT, were performed on the same day. The ratio of the lung volume assessed using single breath methods (alveolar volume; V_A_) to that using multiple breath methods (TLC) was calculated as an index of ventilation heterogeneity. The volume of the pulmonary small vessels <5 mm^2^ in the whole lung (BV5 volume), and number of BV5 at a theoretical surface area of the lungs from the plural surface (BV5 number) were evaluated using chest CT images.

**Results:** The low V_A_/TLC group (the lowest quartile) had significantly lower BV5 number, BV5 volume, higher BV5 volume/BV5 number, and higher Kco compared to the high V_A_/TLC group (the highest quartile) in 117 non-smokers, but not in 67 smokers. Multivariable analysis showed that low V_A_/TLC was associated with low BV5 number, after adjusting for age, sex, weight, lung volume on CT, and CT emphysema index in non-smokers (not in smokers).

**Conclusion:** Ventilation heterogeneity may be associated with low BV5 number and high Kco in non-smokers (not in smokers). Future studies need to determine the dynamic regional system in ventilation, perfusion, and diffusion in asthma.

## 1 Introduction

Single breath methods are used to determine the rate constant of the alveolar uptake of carbon monoxide (CO) for 10 s at barometric pressure, that is, transfer coefficient of the lung for CO (Kco) and alveolar volume (V_A_) (Krogh, 1915; Hughes and Pride, 2012). Kco more sensitively reflects the uptake efficiency of alveolar-capillary units than diffusing capacity of the lung for carbon monoxide (DLco), because DLco is affected by the two components of Kco and V_A_ (Shimizu et al., 2018). Though high Kco is known to be a feature of non-smokers with asthma, the mechanism of increasing Kco is not fully understood. Low forced expiratory flow at 50% of the forced vital capacity (FVC) (FEF50) is associated with increased capillary blood volume when adjusted for V_A_ and high Kco in asthma (Stewart, 1988), which led to a speculation that increased blood volume due to negative intrathoracic pressure causes high Kco. Meanwhile, ventilation heterogeneity has been shown to be strongly correlated with airway hyperresponsiveness (Downie et al., 2007) and asthma symptoms (Farah et al., 2012). To regulate pulmonary perfusion, ventilation heterogeneity should be compensated by the redistribution of blood volume, i.e., decreased blood volume to poorly-ventilated area and increased blood volume to well-ventilated area, for an efficient gas-exchange (Prisk et al., 2020), which is suggested as the theory for high Kco in non-smokers with asthma (Hughes and Pride, 2012).

To infer the nature and degree of the ventilation heterogeneity, the physiological assessment such as plethysmography (Cournand et al., 1941; King et al., 2005), has been utilized, together with radiological assessment, including magnetic resonance imaging (MRI) (Svenningsen et al., 2018), single photon emission computed tomography (Svenningsen et al., 2018), and computed tomography (CT) (Shima et al., 2022). The ratio of V_A_ assessed by single breath methods (Krogh, 1915) to total lung capacity (TLC) (Roberts et al., 1990) assessed by multiple breath methods (V_A_/TLC) has clinical convenience for examining ventilation heterogeneity. V_A_/TLC decreases along with ventilation heterogeneity because V_A_ is more strongly affected by ventilation heterogeneity due to less CO/helium (He) mixture in single breath, compared with TLC measured in multiple breath methods, where CO/He dilutes more broadly (Hughes and Pride, 2012).

The morphological assessment of pulmonary small vessels on CT has progressed from two dimensional (Matsuoka et al., 2010) to three dimensional (Estépar et al., 2013; Ash et al., 2018; Washko et al., 2019), and was confirmed to correlate with cross-sectional vessel dimensions on histology (Rahaghi et al., 2019). The ratio of the aggregate of small vessel cross-sectional area <5 mm^2^ (CSA_<5_) to total lung area (%CSA_<5_) is higher in patients with pulmonary arterial hypertension than that in healthy controls (Shimizu et al., 2017), suggesting that an elevated pulmonary arterial pressure likely increases the number of detectable small vessels, resulting in an increased number of CSA_<5_ and higher %CSA_<5_ value. Contrary to this phenomenon, ventilation heterogeneity may decrease the detectable numbers of CSA_<5_ reflecting the reduction of blood flow/volume in less ventilated areas.

Collectively, we hypothesized that an efficient blood volume regulation, i.e., a reduced blood volume in less ventilated area and an increased blood volume in well ventilated area, contributes to high Kco in patients with asthma. The aim of this study was to examine whether V_A_/TLC is associated with the number of small vessels at a cross-sectional area <5 mm^2^ of theoretical lung surface areas (BV5 number) and the aggregate of blood vessel volumes <5 mm^2^ in the lungs (BV5 volume), in a multi-center prospective asthmatic cohort.

## 2 Methods

### 2.1 Participants

All patients with asthma included in this study had participated in the Hokkaido-based Investigative Cohort Analysis for Refractory Asthma (Hi-CARAT) study, that included individuals with varying levels of asthma severity. From February 2010 to September 2012, patients with asthma were recruited at Hokkaido University Hospital and 29 affiliated hospitals and clinics. The Hi-CARAT study was approved by the Ethics Committee of the Hokkaido University School of Medicine (approval number, 02-001) and registered in the University Hospital Medical Information Network Clinical Trials Registry (UMIN-CTR) system (https://upload.umin.ac.jp/cgi-open-bin/ctr/ctr_view.cgi?recptno; R000003917; ID no. 000003254) (Kimura et al., 2017; Konno et al., 2018). The respiratory physicians made the diagnosis of asthma based on the Global Initiative for Asthma (GINA) criteria (Reddel et al., 2022); the recurrent episodes of respiratory symptoms associated with demonstrable reversible airflow limitation. The definition of severe asthma was based on the American Thoracic Society (ATS) criteria of refractory asthma in 2000 (Proceedings of the ATS workshop, 2000), with a slight modification of the inhaled corticosteroid doses due to its availability in Japan (Konno et al., 2018).

We classified the participants into two groups based on the number of cigarette packs they smoked [non-smokers (<10 pack-years) (N = 117) and smokers (≥10 pack-years) (N = 67)]. Pulmonary function tests followed by CT scan were performed on the same day. Given the severity of asthma in patients, the use of short-acting bronchodilators for at least 12 h, but no other respiratory medications, before obtaining all measurements, was prohibited.

### 2.2 Quantitative chest CT

Participants underwent a multidetector row spiral CT scan with a 64-detector array (Aquilion Multi, TSX-101A/6A; Toshiba Medical Systems, Tochigi, Japan) at the Hokkaido University Hospital (Shimizu et al., 2022). The scan was performed with no contrast during the full inspiration phase. The acquisition parameters were 120 kVp, 300 mA, 64 detectors, 0.5 mm collimation, slice thickness of 0.5 mm, 0.5 s/rotation, helical pitch of 41, and smooth and sharp reconstruction kernels (FC03 and FC52). Vessel and parenchymal analyses were conducted using FC03, while airway analysis was done using FC52.

### 2.3 Assessment of vascular-related, airway, and parenchymal indices

#### 2.3.1 Vascular-related indices including BV5 volume and BV5 number

Quantitative analysis of Digital Imaging and Communications in Medicine (DICOM) data were performed using A-VIEW software (Coreline soft Inc., Seoul, South Korea). In brief, the pulmonary vessels were extracted using a threshold of −750 Hounsfield units (HU) (Jerman et al., 2015; Cho et al., 2018). Beyond Frangi algorithm has been adapted to the software to improve the accuracy of evaluating vasculature (Park et al., 2022). The assessment based on Beyond Frangi algorism was validated by s 3D synthetic image with a grid of 230 × 320×100 voxels with isotropic spacing of 0.5 mm created similar to the shape of (cerebral) angiograms: tubular structures with various radii, bents, bifurcations and aneurysms, together with the fifteen 3D-digitally subtracted angiograms. Compared to other algorism by Frangi et al. (1998); Sato et al. (2000); Li et al. (2003), and λ2 (Wiemker et al., 2013) in the novel algorism by Park et al. (2022) showed the relatively higher area under the precision-recall curve, median of the normalized lter’s response inside the reference segmentation and signal-to-noise ratio. The summed number of small vessels at a cross-sectional area <5 mm^2^ of theoretical lung surface area s at 6 mm from the pleural surfaces in all the lobe (BV5 number) was assessed (Jerman et al., 2015; Cho et al., 2018), while the aggregate blood vessel volume <5 mm^2^ of the total lungs was defined as BV5 volume (17, 25.26). The ratio of BV5 volume to V_A_ (BV5 volume/V_A_) was used to assess the relative blood volume to well-ventilated area, which might reflect the state of the capillary bed and the efficacy of diffusion, especially Kco. Given the presumable increase in blood volume in well-ventilated area, we examined whether the relative ratio of blood volume to detectable blood number (BV5 volume/BV5 number) was increased in heterogeneously ventilated lungs.

#### 2.3.2 Percent wall area (%WA), airway fractal dimension (AFD), and percent low attenuation area (%LAA)

Quantitative assessment of airway dimensions and emphysematous regions was performed using Synapse Vincent (Fujifilm Medical, Tokyo, Japan) (Shimizu et al., 2022). The average of airway luminal area and wall area in the central 1/3 of the branches were measured automatically. Luminal area (LA), wall area percent [the ratio of the wall area to the summed area, between the wall and lumen (%WA) at the right apical (RB1) and lateral basal (RB8) segmental airways], and averaged LAs were normalized by the body surface area (BSA). To quantify a whole airway structure, the AFD was calculated based on the box-counting method, as reported (Bodduluri et al., 2018; Kimura et al., 2022). We adopted a percentage of low attenuation area (%LAA), which is the ratio of the LAA < −950 HU to the lung volume on CT, as an index of emphysema.

### 2.4 Pulmonary function tests

Chestac (Chest MI Inc., Tokyo, Japan) was used for the evaluation of spirometry, DLco, and lung volume. A quality-control protocol was based on the criteria adopted by the Lung Health Study and Japanese Respiratory Society (JRS) guidelines, to improve the accuracy of the examination (Konno et al., 2018). Maintenance and calibration were conducted, in line with the JRS guidelines (Guidelines for pulmonary function tests, 2004).

#### 2.4.1 Spirometry

All measurements of forced expiratory volume in one second (FEV_1_) or FVC were performed using a rolling seal spirometer. Acceptable spirometry measurements required the examinations to be performed in triplicate and greater than two reproducible measurements were mandatory from up to eight forced expirations, in accordance with JRS guidelines. Of those measurements, the best FEV_1_ and FVC were recorded. Furthermore, the acceptability of the spirometric data, using flow volume curves, were assessed by an independent investigator, without any other information on the participants.

Predicted values of spirometric measurements were in accordance with the JRS guidelines on pulmonary function tests. The best FEV_1_ and FVC were recorded, in accordance with the JRS guideline. The pre-bronchodilator values of spirometry are used for the analysis, which are the simultaneous measures with the single breath methods.

#### 2.4.2 DLco, Kco, and V_A_

Patients were required to refrain from smoking on the day of the test for at least 2 h before measurements. DLco was assessed using the single breath method just after pre-bronchodilator spirometry, in a seated position. After rapid inspiration of a gas mixture that comprised of approximately 0.3% carbon monoxide, 10% helium, 21% oxygen, and 68.7% nitrogen, from the residual volume (RV) level, the patients held their breath for approximately 10 s. Alveolar samples were collected and the washout volumes of 750 mL and 500 mL were discarded in patients with vital capacity ≥2.0 and <2.0 L, respectively. Kco, and V_A_ were measured for the analyses. Regarding the correction for hemoglobin concentration, we adopted an equation by ATS and European Respiratory Society guidelines (Reddel et al., 2022). The prediction equation of Burrows was adopted for DLco and Kco (Burrows et al., 1961).

We considered V_A_/TLC as the index of ventilation heterogeneity. Low V_A_/TLC may indicate severe ventilation heterogeneity.

#### 2.4.3 Lung volumes

Lung volumes [TLC, functional residual capacity (FRC), and RV] were assessed using the multiple breaths in helium closed circuit methods. Lung volumes were expressed as percentages of the predicted values according to Nishida prediction equations (Nishida et al., 1961).

### 2.5 Statistical analysis

We divided non-smokers and smokers with asthma based on the V_A_/TLC into three groups: low (lowest quartile), moderate (second and third quartiles), and high V_A_/TLC (highest quartile). For the comparisons of demographics and pulmonary function test values between non-smokers and smokers, Student’s t-test and Wilcoxon signed ran test were used for normally-distributed; log Eosinophil, log IgE, pre%FEV_1_, FEV_1_/FVC, and V_A_/TLC, and non-normally distributed variables; age, height, weight, BMI, duration of asthma, pack-year of tobacco, DLco, %DLco, Kco, %Kco, RVTLC, and %TLC, respectively. Comparing demographics and pulmonary function tests among the three groups (low, moderate, and high V_A_/TLC), ANOVA, followed by Tukey’s multiple comparison test; Dunn’s test for continuous variables, and chi-square test for categorical variables, were used. As for CT indices, Dunnett’s test was used, taking high V_A_/TLC group as controls. The correlation of V_A_/TLC and morphological (CT-based) indices such as BV5 number, BV5 volume, BV5 volume/V_A_, BV5 volume/BV5 number, %LAA, %WA, and AFD, was examined using Pearson and Spearman correlation coefficients. Multiple regression analysis was performed to evaluate the relationships of BV5 number, BV5 volume with V_A_/TLC including sex (as a categorical variable), age, weight, lung volume on CT (Wu et al., 2022), %LAA, and %WA. All analyses were performed using JMP 16.0.0 (SAS Institute, Cary, North Carolina, United States).

## 3 Results

### 3.1 Characteristics of the participants

**FIGURE 1:**
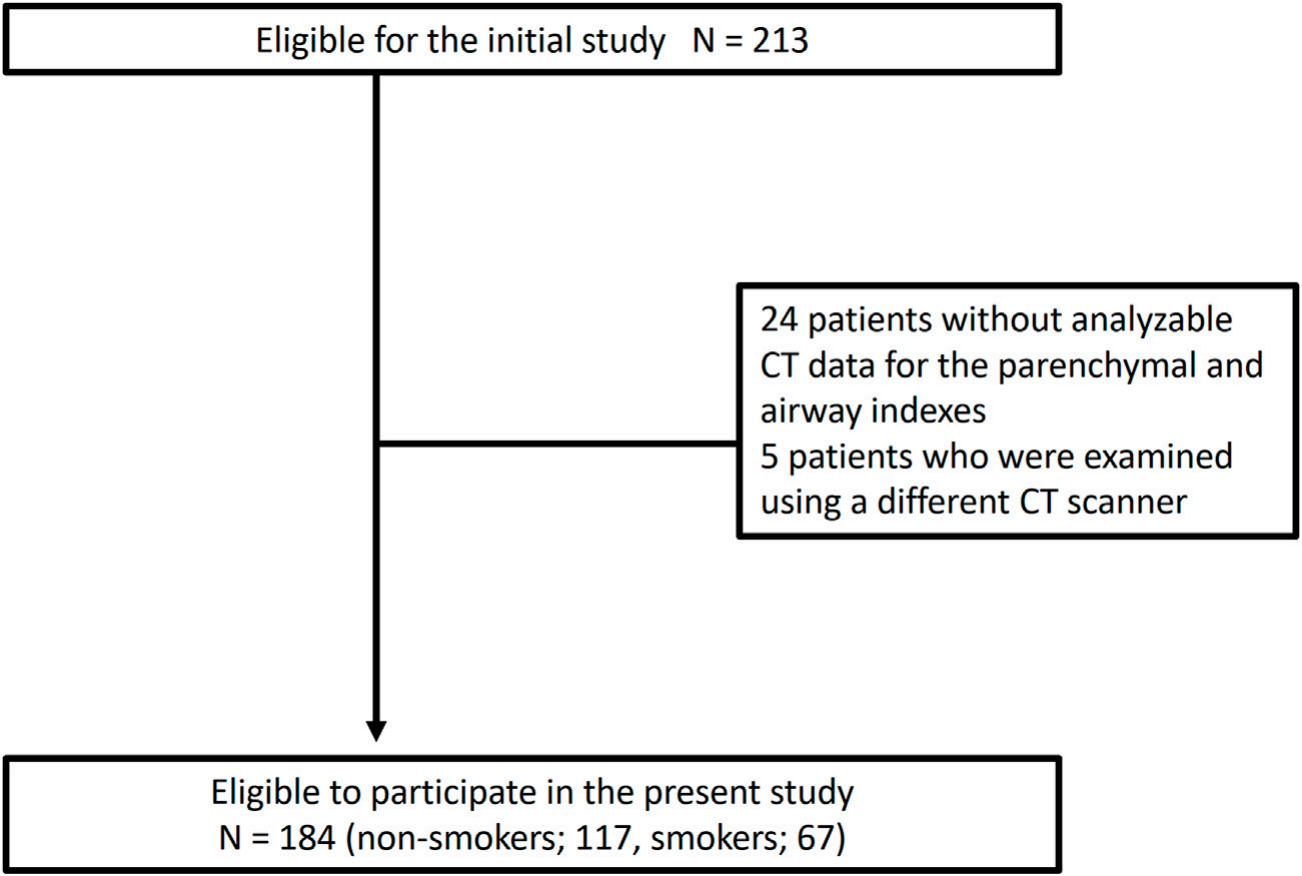
Flow chart of participants Of 213 patients in the initial study, 24 patients did not have analyzable CT data for pulmonary vessels, airway, and parenchymal indexes, five patients were examined using a different CT scanner. Finally, 184 patients were included for further analysis.

Among 213 patients enrolled in the Hi-CARAT study, 24 patients did not have the analyzable CT data for BV5 number, BV5 volume, %WA, AFD, and %LAA, and five patients underwent CT scan that was obtained using different scanners from the other patients, then 184 patients with asthma of 117 non-smokers (male *n* = 25/female *n* = 92) and 67 smokers (male *n* = 20/female *n* = 47) were included (Figure 1, Table 1). Weight and height were higher in smokers with asthma compared with non-smokers with asthma. %FEV_1_, FEV_1_/FVC, Kco, and %Kco were lower in smokers with asthma compared with non-smokers with asthma, while DLco, V_A_/TLC, RV/TLC, and %TLC were not different between the two groups.

**TABLE 1:** Characteristics of the participants according to the V_A_/TLC categories in non-smokers and smokers.

	All	Non-smokers				Smokers			
			Low V_A_/TLC	Mod V_A_/TLC	High V_A_/TLC		Low V_A_/TLC	Mod V_A_/TLC	High V_A_/TLC
**N**	184	117	29	59	29	67	17	34	16
**Age** ^b^	60.3 (13.2)	60.5 (14.0)	67.3 (8.8)	60.5 (14.1)	53.9 (15.2)	61.6 (11.7)	61.4 (13.3)	63.9 (10.5)	56.9 (11.6)
**Female, % (N)** ^a^ ^,^ ^b^	60.9 (112)	82,1 (92)	97.0 (28)	78.0 (47)	62.0 (18)	29.9 (20)	41.2 (7)	23.5 (8)	31.3 (5)
**Weight, kg** ^a^ ^,^ ^b^	62.4 (14.1)	60.9 (13.0)	56.0 (7.8)	60.2 (14.7)	67.1 (11.2)	65.3 (14.5)	66.5 (22.0)	62.7 (13.2)	69.5 (11.8)
**Height, cm** ^a^	157.8 (9.0)	155.9 (7.8)	151.0 (4.7)	155.4 (7.4	162.0 (7.4)∗	161.1 (10.1)	159.6 (10.2)	161.2 (10.1)	161.6 (10.4)
**BMI, kg/m** ^**2**^	25.1 (5.1)	25.0 (4.8)	24.6 (3.7)	24.6 (3.7)	25.6 (3.9)	25.2 (5.5)	26.3 (8.2)	23.9 (4.3)	26.57288
**Severe, % (N)**	65.8 (121)	61.5 (72)	62.1 (18)	61.0 (36)	62.1 (18)	73.1 (49)	76.5 (13)	70.6 (24)	75.0 (12)
**Duration of asthma, yo**	19.8 (8–28.8)	20.7 (9–29)	18 (10–28.5)	21.0 (8–35)	12 (8.5–25)	18.3 (7–25)	17 (8.5–21)	13.5 (7–25)	17.5 (15.7)
**Pack-years** ^a^	13.2 (0–18.7)	1.42 (10–2.2)	0.0 (0.0–0.0)	0.0 (0.0–0.7)	0.0 (0.0–5.3)	33.7 (15.6–46)	27.5 (16.6–58)	30 (14.3–48)	20.5 (13–30)
**Eo,/μL**	323 (111–446)	326 (109–468)	270I136-610)	250I90-476)	191I137-428)	318 (112–433)	386 (152–628)	167 (105–380)	174 (70–312)
**IgE, IU**	421 (59–444)	412 (55–345)	125 (64–302)	170 (47–370)	170 (80–362)	185.6 (65–576)	319 (121–709)	195 (59–471)	236 (37–481)
**pre%FEV**_**1**_ ^a^ ^,^ ^b^	86.6 (19.9)	89.2 (19.6)	76.1 (17.0)	91.6 (18.6)	97.4 (18.1)	82.1 (19.8)	75.3 (20.7)	81.2 (20.1)	91.4 (15.5)
**preFEV**_**1**_ **/FVC, %** ^a^ ^,^ ^b^	64.6 (12.6)	66.2 (12.2)	60.4 (11.6)	66.7 (12.3)	70.9 (10.4)	61.7 (12.9)	58.6 (15.5)	60.6 (12.2)	.67.5 (10.0)
**DLco, mmol/min/mmHg** ^b^ ^,^ ^c^	18.6 (4.47)	18.3 (4.2)	16.0 (2.2)	18.3 (4.1)	20.7 (4.6)	19.0 (4.9)	16.9 (4.5)	19.0 (5.2)	21.3 (4.0)
**%DLco, %**	104.1 (22.2)	103.6 (20.7)	101.9 (18.5)	105.3 (20.9)	101.9 (22.8)	105.0 (24.8)	93.9 (26.2)	109.5 (25.3)	107.1 (19.4)
**Kco, ml/min/mmHg/L** ^a^ ^,^ ^b^	5.1 (1.0)	5.4 (0.9)	5.8 (1.1)	5.3 (0.8)	5.0 (0.7)	4.6 (1.0)	4.5 (1.0)	4.5 (1.1)	4.9 (0.6)
**%Kco, %** ^a^ ^,^ ^b^	109.0 (22.1)	114.7 (20.9)	130.7 (25.5)	112.7 (16.4)	102.7 (13.6)	99.0 (20.5)	97.6 (21.2)	98.3 (22.5)	102.2 (15.6)
**V**_**A**_ **/TLC, %** ^b^ ^,^ ^c^	72.8 (4.8)	72.9 (4.8)	66.5 (2.3)	73.0 (1.8)	79.0 (1.9)	72.8 (4.7)	66.6 (3.4)	73.4 (1.7)	78.1 (1.9)
**RV/TLC, %** ^b^ ^,^ ^c^	36.4 (6.9)	36.1 (7.0)	43.0 (4.7)	35.8 (5.4)	29.8 (5.2)	36.8 (6.8)	41.0 (5.9)	37.0 (6.8)	32.0 (4.5)
**%TLC, %**	112.7 (13.6)	112.3 (13.5)	108.9 (13.6)	113.4 (13.4)	113.4 (13.4)	113.5 (13.8)	117.4 (15.8)	112.2 (12.4)	112.2 (14.2)

Data are shown as the mean ± standard deviation (SD), median (interquartile range), geometric mean (log10 SD), or number (%).

BMI, body mass index; Eo, blood eosinophil count; pre%FEV_1_, pre-bronchodilator percentage of predicted orced expiratory volume in 1 s; preFEV_1_/FVC, pre-bronchodilator FEV_1_/forced vital capacity; RV, residual volume; DLco, diffusing capacity for carbon monoxide; Kco, carbon monoxide transfer coefficient; V_A_, alveolar volume; TLC, total lung capacity.

a Significant difference between non-smokers with asthma vs. smokers with asthma.

b Significant difference among groups with low, moderate, and high V_A_/TLC, in non-smokers.

c Significant difference among groups with low, moderate, and high V_A_/TLC, in smokers.

**FIGURE 2:**
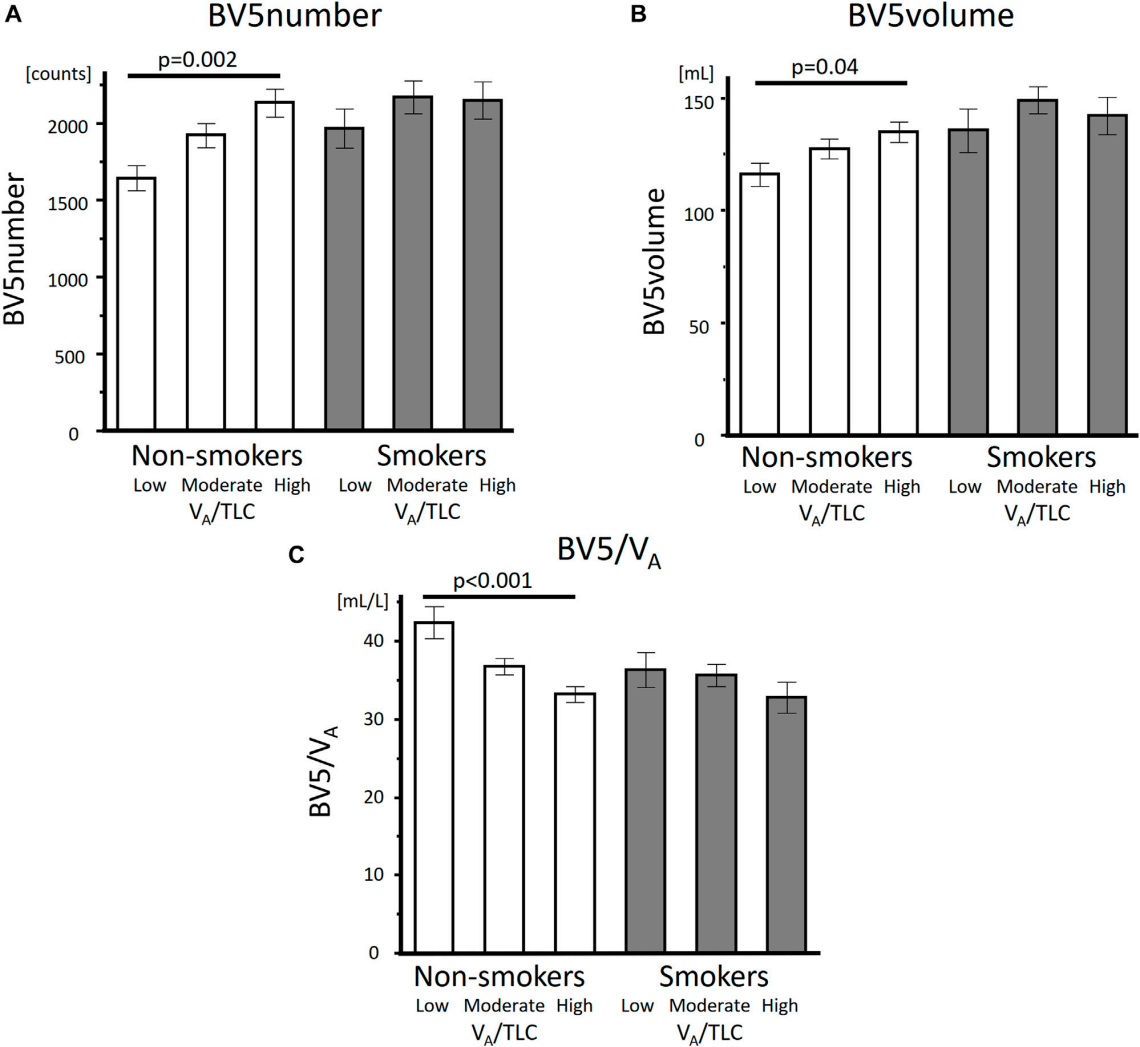
Comparisons of BV5 number, BV5 volume, and BV5 volume/VA among the low, moderate, and high VA/TLC groups in non-smokers with asthma and smokers with asthma BV5 number **(A)**, BV5 volume **(B)** were lower and BV5 volume/VA **(C)** was higher in low VA/TLC group than those in the high VA/TLC group in non-smokers with asthma, but not in smokers with asthma.

**FIGURE 3:**
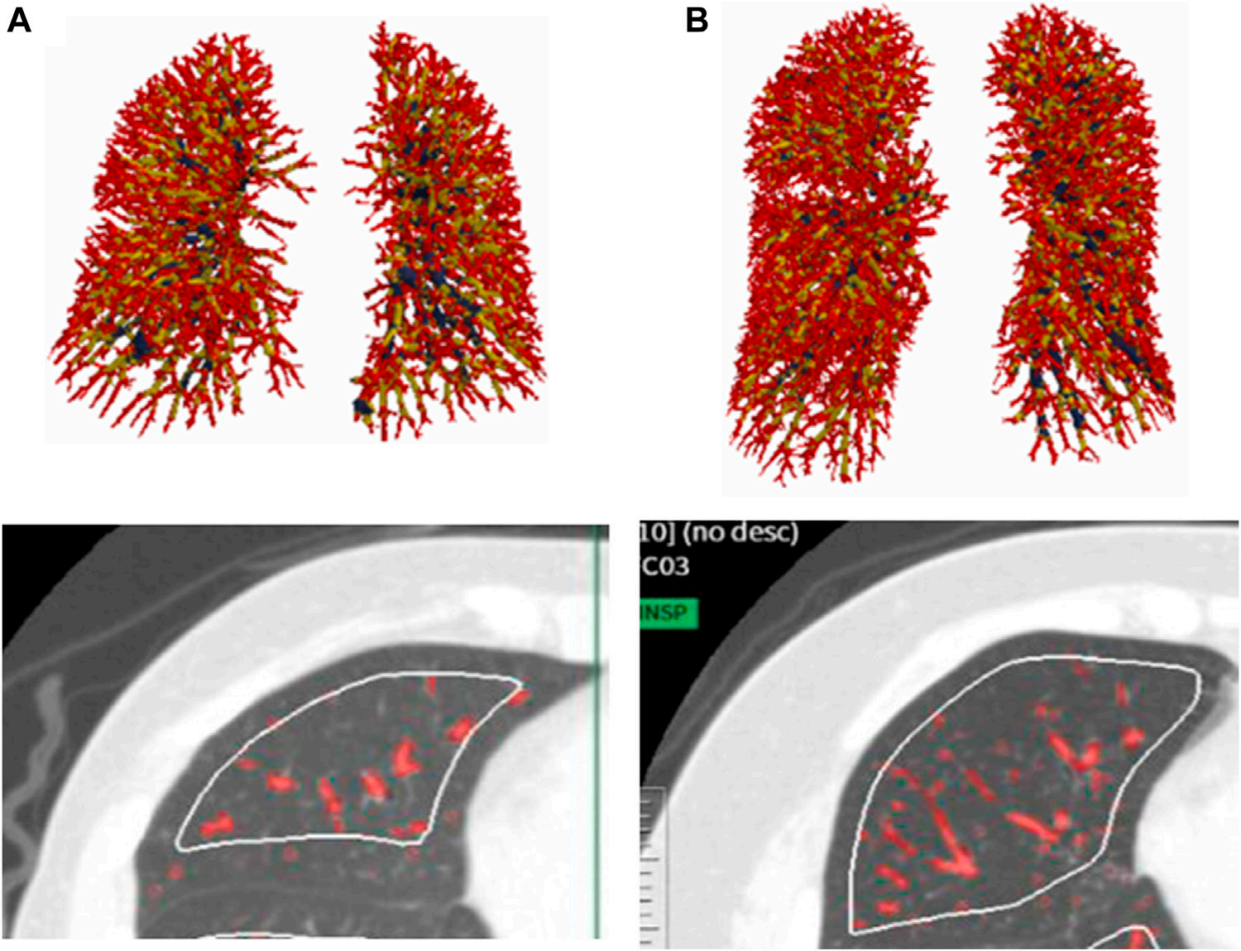
Representative three-dimensional imaging and axial CT images in two female patients. One with low VA/TLC (60.9%) had 1943 counts of BV5 number, and 109.8 mL of BV5 volume **(A)**, while another with high VA/TLC (83.%) had 2812 counts of BV5 number, and 156 mL of BV5 volume **(B)**.

As for the comparison of the groups based on V_A_/TLC, patients in low V_A_/TLC group were older and female dominant. In non-smokers with asthma, %FEV_1_, FEV_1_/FVC, DLco, BV5 number, and BV5 volume, were significantly lower in low V_A_/TLC group than in high V_A_/TLC group (Table 1; Figure 2). Kco and %Kco, RV/TLC, BV5 volume/V_A_ were significantly higher in low V_A_/TLC group than those in high V_A_/TLC group. As for smokers with asthma, there were no significant differences in BV5 number, BV5 volume, %Kco, and BV5 volume/V_A_, but DLco was lower, RV/TLC was higher in low V_A_/TLC group than those in high V_A_/TLC group. Figure 3 shows representative three-dimensional imaging and axial CT images in two female participants. One with low V_A_/TLC (60.9%) had 1943 counts of BV5 number, and 109.8 mL of BV5 volume (A), while another with high V_A_/TLC (83.3%) had 2812 counts of BV5 number, and 156 mL of BV5 volume (B).

### 3.2 Univariable analysis of morphological CT-based indices and V_A_/TLC

#### 3.2.1 Non-smokers with asthma

%LAA was .88 (.11–.81); median (interquartile range). %WA and AFD were 59.2 (7.5), 1.93 (.06); average (standard deviation), respectively. %LAA is Low V_A_/TLC significantly correlated with high %WA, but there was no significant correlation between V_A_/TLC and %LAA (Table 2).

**TABLE 2:** Relationships of morphological indices with V_A_/TLC.

	Non-smokers	Smokers
%WA	R = −0.34, *p* < 0.001	R = −0.29, *p* = 0.02
AFD	R = 0.34, *p* < 0.001	R = −0.09 *p* = 0.48
%LAA	R = −0.03, *p* = 0.72	R = −0.30, *p* = 0.01

FEV_1_, forced expiratory volume in 1 s; FVC, forced vital capacity; WA, wall area; AFD, airway fractal dimension; %LAA, the percentage of low attenuation area.

#### 3.2.2 Smokers with asthma

%LAA was 2.75 (.16–2.39); median (interquartile range). %WA and AFD were 58.8 (6.0), 1.94 (.06); average (standard deviation), respectively. Low V_A_/TLC significantly correlated with airway disease (high %WA) and parenchymal disease (%LAA) (Table 2).

### 3.3 Relationships of V_A_/TLC with BV5 number and BV5 volume, and with BV5 volume/BV5 number

#### 3.3.1 Non-smokers with asthma

**FIGURE 4:**
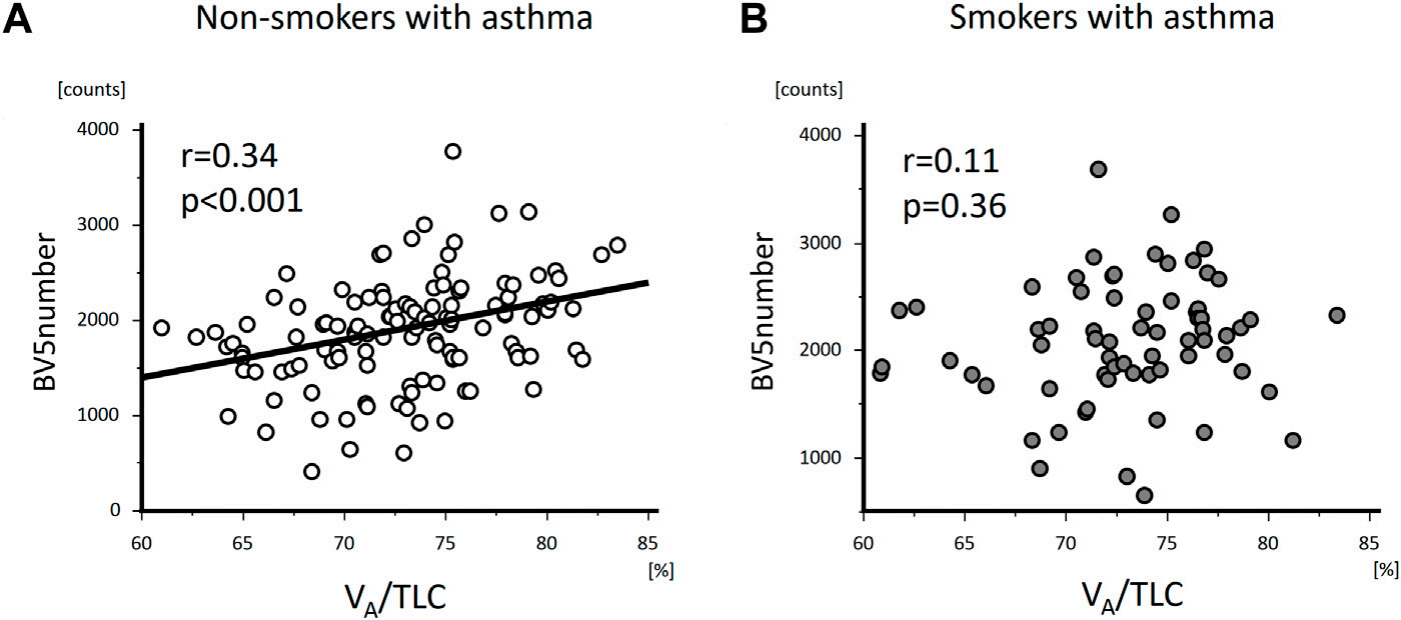
The relationships of VA/TLC with BV5 number in non-smokers and smokers with asthma. VA/TLC was positively associated with BV5 number in non-smokers with asthma **(A)**, but not in smokers with asthma **(B)**.

**FIGURE 5:**
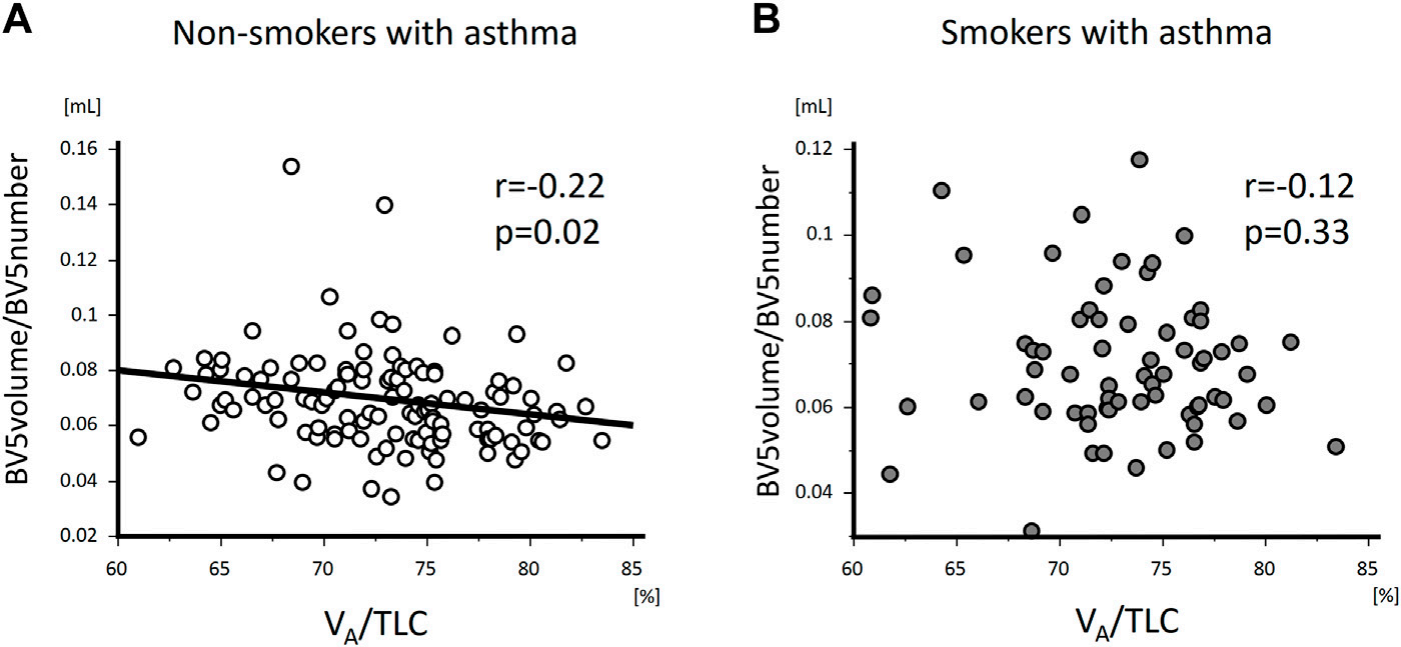
The relationships of VA/TLC with BV5 volume/BV5 number in non-smokers and smokers with asthma VA/TLC was negatively associated with BV5 volume/BV5 number in non-smokers with asthma **(A)**, but not in smokers with asthma **(B)**.

V_A_/TLC was positively associated with BV5 number and negatively with BV5 volume/BV5 number (Figure 4 and Figure 5) Multivariable analysis showed that low V_A_/TLC correlated with low BV5 number, but not low BV5 volume, independent of age, sex, weight, lung volume on CT, %LAA (Table 3 and Table 4).

**TABLE 3:** Relationships of V_A_/TLC with BV5 number in non-smokers and smoker with asthma (Multivariable anaiysis).

	Non-smokers		Smokers	
	Estimate (95% C.I.)	p	Estimate (95% CI)	p
V_A_/TLC	20.3 (3.7–37)	0.02	14.3 (−-16.7–45.3)	0.36
Age	−14.2 (−20.7–−7.7)	<0.001	−9.0 (−21.5–3.6)	0.16
Male	−18.5 (−131.0–94.0)	0.75	−54.1 (−255.1–146.8)	0.59
Weight	−13.8 (−19.8–7.9)	<0.001	−7.5 (−17.4–2.4)	0.13
CT-LV	0.36 (0.23–0.49)	<0.001	0.21 (0.006–0.41)	0.044
%LAA	−212.3 (−360.0–64.6)	0.005	−173.4 (−440.3–93.5)	0.20

V_A_, alveolar volume; TLC, total lung capacity; CT-LV, lung volume assessed on CT; %LAA, the percentage of low attenuation area; %LAA, were log10 transformed C.I, confidential interval.

**TABLE 4:** Relationships of V_A_/TLC with BV5 volume in non-smokers and smokers with asthma (Multivariable analysis).

	Non-smokers		Smokers	
	Estimate (95% CI)	p	Estimate (95% CI)	p
V_A_/TLC	0.29 (−0.73–1.32)	0.57	0.80 (−0.91–2.51)	0.35
Age	−0.35 (−-0.75–0.04)	0.08	−0.73 (−1.4–0.03)	0.04
Male	1.67 (−5.26–8.61)	0.63	2.4 (−8.6–13.6)	0.66
Weight	−0.42 (−0.79–0.06)	0.02	0.08 (−0.47–0.62)	0.77
CT-LV	0.02 (0.01–0.03)	<0.001	0.02 (0.006–0.028)	0.003
%LAA	−8.8 (−17.9–0.33)	0.06	−0.76 (−15.5–14.0)	0.35

V_A_, alveolar volume; TLC, total lung capacity; CT-LV, lung volume assessed on CT; %LAA, the percentage of low attenuation area; %LAA, were log10 transformed; C.I, confidential interval.

#### 3.3.2 Smokers with asthma

V_A_/TLC did not correlate with BV5 number or BV5 volume/BV5 number (Figure 4 and Figure 5), as was the case with the multivariable analysis on the relationship with BV5 number or BV5 volume (Table 3 and Table 4).

## 4 Discussion

This morphological study demonstrated that low V_A_/TLC was associated with a reduction in pulmonary small vessel number (BV5 number), volume (BV5 volume), an increase in BV5 volume/BV5 number, and Kco in non-smokers with asthma. These may suggest that the number of detectable small blood vessels decreases and the volume of detectable small vessels increases as ventilation heterogeneity progresses, which is in harmony with the physiological theory for the redistribution of blood volume to match the ventilation-perfusion, followed by high Kco in intact alveolar-capillary units in non-smokers with asthma. Whereas low V_A_/TLC did not show any association with BV5 number, BV5 volume, or Kco in smokers with asthma.

Ventilation heterogeneity represented by V_A_/TLC was related to decreased BV5 number after adjusting for age, sex, lung volume on CT (Wu et al., 2022), and emphysema index in non-smokers with asthma. Moreover, the ratio of BV5 volume to BV5 number increased as V_A_/TLC decreased, which might suggest an increased blood volume in the well-ventilated area. Thus, those alterations of CT-based vasculature may support the redistribution of blood volume for ventilation-perfusion matching in non-smokers with asthma. Stewart showed that a high ratio of pulmonary capillary blood volume to lung volume assessed by the single breath methods was related to a high Kco in asthma with mild airflow limitation (Stewart, 1988), which is concordant to a high BV5 volume/V_A_ and %Kco in the low V_A_/TLC group, in the current study. Increased blood flow to the apex of the lungs assessed by an increased Xe uptake in the upright position was reported as an associated factor for high Kco in non-smokers with asthma (Collard et al., 1994). A regional (Sato et al., 2015) and dynamic simultaneous assessment of pulmonary circulation and ventilation with high resolution under the patient being in an upright position, would totally unveil the linkage of ventilation, perfusion, and diffusion in heterogeneously ventilated lungs of non-smokers with asthma.

Of note, low V_A_/TLC correlated with neither high Kco, low BV5 number, nor low BV5 volume in smokers with asthma. It is presumed that emphysematous lungs fail to compensate the diffusion *via* the redistribution of blood volume (Hughes and Pride, 2012). Though the patients with asthma in the current study had limited evidence of emphysema on CT, smoking may cause the dysfunction of the alveolar capillary units, which might be linked to a decreased vascular endothelial growth factor in the bronchoalveolar lavage fluid (Nagai et al., 2005). A dynamic CT study showed more prominent heterogeneity of the mean transient time and the pulmonary blood flow was identified in smokers with subtle centrilobular emphysema, compared with non-smokers or smokers with no emphysema on CT (Alford et al., 2010), which might imply that the vascular dysfunction led to a failure of V/Q match; this should be fully explored in a future study.

BV5 volume and BV5 number have differential roles in assessing a distribution of blood volume on CT. BV5 number is the number of vessels <5 mm^2^ in the theoretical lung surface area at 6 mm depths from the pleural surface, while BV5 volume is equivalent to the total volume of vessels <5 mm^2^ in the entire lungs. The maldistribution of ventilation causes an increase in the blood volume in well-ventilated areas and a decrease in poorly-ventilated areas, simultaneously, leading to the relatively preserved BV5 volume. It is presumed that a low number of small vessels may reflect the reduction in the blood volume at the level of the alveolar capillary unit more sensitively, compared with that of BV5 volume. The novel modality, which enables the combined assessment of ventilation and perfusion (Wang et al., 2021) beyond the resolution of CT, would further broaden the insight into the micro hemodynamics in the lungs.

Low V_A_/TLC correlated with low %FEV_1_ and FEV_1_/FVC both in non-smokers and smokers with asthma. However, the differential associations of CT-based morphological indices with V_A_/TLC were suggested between non-smokers and smokers with asthma in this study. %WA at the segmental generation and AFD, but not %LAA, were related to V_A_/TLC in non-smokers with asthma, while %WA, %LAA, but not AFD, were related to V_A_/TLC in smokers with asthma. AFD reflects the morphological complexity of the whole bronchial tree on CT, a decrease of which is caused by airway narrowing due to the remodeling of the airway or secretion/mucus in the airway (Bodduluri et al., 2018). All proximal airway diseases may affect ventilation heterogeneity in non-smokers with asthma, which is concordant with previous reports (Foy et al., 2020).

To understand that the relationship of ventilation heterogeneity with gas-exchange is of clinical use in the management of respiratory diseases (Neder et al., 2019; Stanojevic et al., 2022). The value of DLco, which is composed both of V_A_ and Kco, does not specify the state of ventilation or gas-exchange. This study confirms the significance of combined interpretation of V_A_ and Kco, suggesting that reduced V_A_ leads to increased Kco in the lungs with no distinct alveolar-capillary damage, *vice versa*, a reduced Kco combined with reduced V_A_ implies the existence of alveolar-capillary damages.

There are several limitations in this study. First, this was not a large-scale study. However, the participants cut across the disease severity categories in both non-smokers and smokers with asthma. Therefore, the findings may be generalized. Second, BV5 volume is the total of the blood volume <5 mm^2^ in the lungs, while the BV5 number was not the total count of BV5 in the whole lungs, but that of the vasculature <5 mm^2^ on CT at the theoretical surface area 6 mm from the pleural surface in all the lobes. Nevertheless, for differences in indices, both BV5 number and BV5 volume may relatively reflect the features of the whole lung. Third, the patients were in the supine position during the CT scan, while they were in the upright position during the pulmonary function tests; therefore, the differential postures may affect the results. A novel modality, such as radiological examination, where the patients are in the upright position would ultimately solve this issue of radiological structure-function study. Fourth, this study assessed the relationship of ventilation heterogeneity assessed by pulmonary function tests with vasculature on CT in the whole lung. Thus, the regional micro-network on ventilation-perfusion match should be explored to broaden our insight into ventilation and the corresponding blood redistribution, which might lead to one of the novel interventions for hypo-oxygenation. Fifth, according to the quantitative analysis of pulmonary vessels, the cut-off value of −750HU to extract vasculature on volumetric CT should be validated by future studies.

In conclusion, CT-based morphological vascular assessment showed a smaller number of detectable small vasculature and increased blood volume for the given V_A_ (BV5 volume/V_A_) or vessel on CT (BV5 volume/BV5 number) and high diffusion in heterogeneously-ventilated lungs of non-smokers with asthma. Advanced methodology is required for the comprehensive knowledge of the intact regulation among respiratory organs and disruption of that system.

## Data Availability

The original contributions presented in the study are included in the article/Supplementary materials, further inquiries can be directed to the corresponding author.
